# In-situ visualization of multiple filament competition dynamic during nonlinear propagation of femtosecond laser

**DOI:** 10.1038/s41598-017-10768-2

**Published:** 2017-09-04

**Authors:** Pengfei Qi, Lie Lin, Qiang Su, Nan Zhang, Lu Sun, Weiwei Liu

**Affiliations:** Institute of Modern Optics, Nankai University, Key Laboratory of Optical Information Science and Technology, Ministry of Education, Tianjin, 300071 China

## Abstract

In this work, multiple filamentation competition of femtosecond pulse in methanol is studied both experimentally and numerically. The visualization of multiple filamentation competition has been realized in the experiment performing the three-photon fluorescence of Coumarin 440. The random changes of multiple filamentation stemmed from the jitter of the peak laser intensity ratio of initial hot spots are first observed directly and visually, which can be well explained by a simplified (2D+1)-dimensional model.

## Introduction

The filamentation is the result of the dynamic counteraction of the optical Kerr effect induced self-focusing and the defocusing effect of the self-generated plasma^1–5^. It has stimulated a lot of research interest because of many promising applications such as remote sensing^6, 7^, lightning control^8, 9^, generation of ultra-broadband light^10–13^ and laser-induced water condensation in the past few years^14, 15^. In the real experimental condition, smooth beam profile with only one intensity maximum is difficult to realize. Hence, multiple filaments are often observed when the laser peak power is higher than the critical power for self-focusing^16, 17^. The underlying physical mechanism of multiple filamentation is due to the non-uniform wave front^18^ by inherent imperfection of the laser or the external perturbation such as turbulence in air^19, 20^ or the passage through an optical component, etc. These filaments are not independent as a result of the competition of energy from the whole pulse’s background reservoir into their own self-foci^3, 21^.

The ‘natural’ formation of multiple filaments is a universal random process, thus there exists the challenge of controlling random processes of multiple filamentation. Various controlling methods by imposing strong modulation onto the initial beam transverse intensity distribution or phase front such as employing pinhole^22^ mesh, phase plate^23, 24^ or axicon^25, 26^ etc. have been reported both theoretically and experimentally. It is worth noting that the directed and visualized observation of the fundamental physics govern the nonlinear propagation of ultrashort pulses and filamentation is indispensable for the controlling and developing applications of multiple filamentation^27^. Due to the high intensity in the filament, direct and precise measurement of the intensity distribution is difficult. In 2004, the fluorescence signal from the excitation of nitrogen molecules inside the plasma channel has been employed to reveal the complex dynamics of multiple filaments propagation^21^. Subsequently, multiple filamentation of collimated beams in air is investigated for beam powers reaching several terawatts by using a white screen positioned in the plane orthogonal to the beam path^28^. The interaction of two light filaments propagating in air has also been reported, which shows that a long and stable channel can be formed by fusing two in phase light filaments^29^. Moreover, with the report of the concept of optical rogue waves, the statistics of light amplitude fluctuations for the propagation of a laser beam subjected to multiple filamentation are investigated extensively, which reveal a non-Gaussian behavior, with long tails corresponding to rogue events^30, 31^.

However, the investigations of the competition of multiple femtosecond laser filaments mentioned above are mainly based on the fluorescence spectra, transverse distribution and theoretical models. To our knowledge, direct and visual observation of multiple filamentation competition has not been reported yet, which provides insight into the physical implication and applications of multiple femtosecond laser filaments. Combined with multiple photon florescence technique, the liquid provided a visible platform to investigate multiple filaments in a single-shot pulse since the filament length varies from several hundred microns to several centimeters depending on the experimental conditions and the dye can be stably and homogeneously dispersed in it^32^.

In this work, the dye solution which contains a very dilute (~0.13%, which has negligible effect on the overall filamentation dynamic^32^) solution of Coumarin 440 in methanol is chose as the propagation medium to demonstrate the competition among the multiple filaments. Direct visualization of the dynamic processes of filamentation competition including filaments interaction and fusion has been observed in our experiment. In the experiment, the captured visible multiple filaments images come from the fluorescence of the dye molecules excited by three photon excitation in the high pulse intensity zones. Meanwhile, it is observed that the random changes of multiple filamentation from shot to shot because of the power and corresponding ratio perturbations of input beam, which can be well reproduced by a simplified (2D+1)-dimensional model.

## Experimental setup

**Figure 1:**
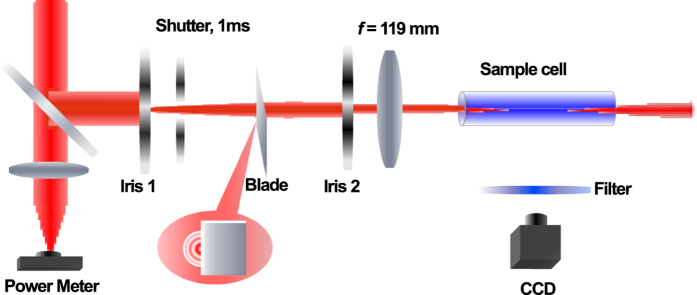
Experimental setup for the visual observation of multiple filamentation competition.

The experimental setup is schematically shown in Fig. 1. The laser pulses with the central wavelength of 800 nm and the pulse duration of 42fs in our experiment are obtained by the commercial CPA femtosecond laser system (Spectra Physics). The laser beam diameter is 5 mm at 1/e^2^ of the maximum energy. A half wave plate located between the laser and the compressor is adopted to modulate the output power. The laser power fluctuation is controlled for less than 3% in our experiments.

After the compressor, the reflection from a beam splitter (R:T = 1:5.6) passed through two irises for spatial filtering, a shutter and a blade before getting to a lens. The iris 1 with 0.6 mm aperture diameter is set 100.2 cm away from the lens, while the distance between the two irises is 92.6 cm. The iris 2 is selected for filtering out the high order diffractions in our optical path. With the help of the spatial filters, the beam with smooth transverse distribution corresponding to the central Airy pattern is formed after iris 2. The blade is adopted to produce the expected location controllable hot spots, which are the origin of multiple filaments. The shutter between the two irises with 1 ms exposure time is selected to allow only one laser pulse during each opening for our laser repetition rate. The shutter works in a single shot mode. A lens with the focal length being 119 mm is set behind the second iris to focus the laser pulse into a glass cell. The distances from the second iris to the lens and from the lens to the cell entrance window were 7.6 cm and 7.1 cm, respectively.

The glass cell with the length 10 cm and the diameter 2 cm has an entrance window and an exit window both of 1 mm thick. The glass cell contained a very dilute solution of Coumarin 440 in methanol. At the side of the glass cell, a narrow bandwidth filter was placed to separate different colors of the generated super-continuum in the experiments. A CCD camera is set to record the transmitted beam pattern from the side of the glass cell. In the whole process of our experiment, a power meter is set behind the beam splitter to calibrate the energy of the beam going into the water cell.

## Experimental Results and Discussion

**Figure 2:**
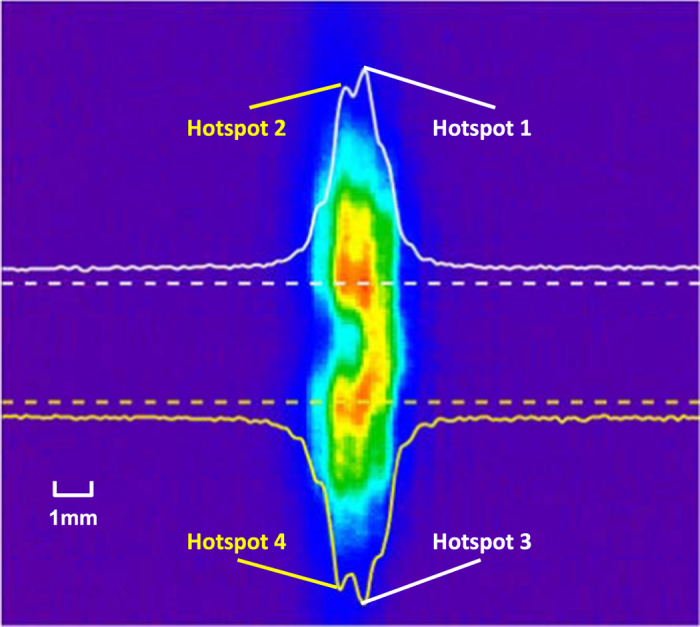
The input beam with four hot spots divided into two pairs, the distance *d* between the peak intensity of the two pairs of hot spots is almost 3 mm, which is no more than 0.5 mm for the two hot spots in a pair. The white and yellow solid curves show the profile of the fluence distribution along the corresponding dotted lines, respectively.

In order to get coherent multiple filaments during the nonlinear propagation of femtosecond laser pulse, the blade for straight edge diffraction and the two irises for spatial filtering are assembly employed to produce the expected more than one hot spots across the beam profile. In our experiment, the input laser peak power is set at 1.4 times threshold power (~6 MW), and the input beam with four hot spots divided into two pairs is obtained by adjusting the blade and irises, as shown in Fig. 2. The white and yellow solid curves show the profile of the fluence distribution along the corresponding dotted lines, respectively, and the hotspots are noted as hotspots 1–4. The distance *d* between the peak intensity of the two pairs of hot spots is larger than 3 mm, which is no more than 0.5 mm for the two hot spots in a pair. It can be predicted that the two hot spots of each pair in the initial beam profile will evolve in two interacting filaments, while the hot spots between the two pairs don’t compete with each other because of the long distance.

**Figure 3:**
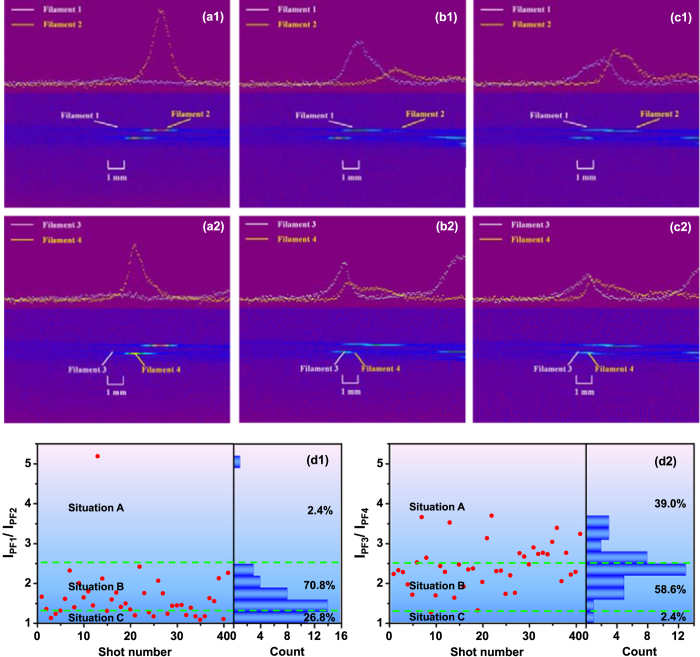
(**a1**–**c1**) and (**a2**–**c2**)The different situations of the interaction of the filaments correspond to two pairs of hotspots during the propagation at three distinct times: the undersides show the CCD images obtained in our experiments and the upsides refer to the relative intensity distributions of the two filaments. (**d1**) and (**d2**) The statistical analysis of the filamentation competition: the filamentation competition is divided into three situations A–C corresponding to (**a1**–**c1**) and (**a2**–**c2**), the dividing lines of three situations are sketched as green dash lines *I*_*PF1,3*_/*I*_*PF2,4*_ = 1.3 and 2.5 and the probabilities of three situations are noted in corresponding regions.

Owing to the fluorescence from dye molecules excited by three photon excitation in the high intensity zones, the competition of multiple filaments can be recorded directly and visually by CCD in experiments as shown in Fig. 3. Moreover, the relative intensity distributions of the femtosecond laser filaments are also characterized by employing the convenient digital image processing technology. The undersides in Fig. 3 show the CCD images obtained in our experiments and the upsides refer to the relative intensity distributions of the two filaments.

The recorded visible filaments changed randomly from shot to shot, as shown in the Supplementary Movie 1 and 2. Firstly, the two filaments corresponding to the two hot spots of the upper pair in Fig. 2 are discussed. Fig. 3(a1–c1) depict the three different situations of the two filaments at three distinct times in the movie 1, respectively. It can be seen that the filament 1 almost disappeared while the filament 2 extended its length to about 3 mm and became mature from Fig. 3(a1). From the intensity profiles of filaments in the upper of Fig. 3(a1), it can be found that the intensity of filament 1 is nearly zero which is much lower than the filament 2. This is because the energy exchange between filaments and background reservoir. To be specific, most of the pulse energy is contained in the large background of the beam, the filament which “suck” the energy from the background energy reservoir will go mature and long, while the other filament that lose the energy will go short or die off gradually. A mature filament is one that has undergone the full processes of self-focusing and filamentation during propagation and will end with self-steepening resulting in the strong spectral broadening (white light laser or supercontinuum). However, the contrasting relationship between the two filaments went the opposite at another time. In Fig. 3(b1), the most noticeable change of the two filaments is that the length of filament 1 is about 2.5 mm which is much longer than the filament 2 (less than 1 mm). Compared to filament 1, filament 2 just looks like a “child” whose width of the peak at 1/*e* ^2^ intensity is also smaller than filament 1. Besides, it can be also observed that the situation that the length of two filaments was almost same, looked like they fused to one filament during the propagation in our experiments as shown in Fig. 3(c1). This is due to the condition of the two filaments with the energy close to each other. When the two filaments meet, they become fusion during the propagation.

In order to quantitatively reveal the complex dynamics of multiple filament propagation, the statistical analysis of the filamentation competition events is depicted in Fig. 3(d1), where the higher peak fluorescence signal intensity and the other one of the filaments 1 and 2 are denoted as *I*_*PF1*_ and *I*_*PF*2_ in the statistic analysis, respectively. Based on the ratio of the peak fluorescence signal intensity between filaments 1 and 2, the filamentation competition events can be objectively divided into three situations A–C corresponding to Fig. 3(a1–c1), namely *I*_*PF1*_/*I*_*PF2*_ > 2.5, 1.3 < *I*_*PF1*_/*I*_*PF2*_ < 2.5 and 1.0 < *I*_*PF1*_/*I*_*PF2*_ < 1.3, respectively. Regions A–C have been considered as only one filament exists, two filaments exist but one is mature and both filaments are mature, respectively. As indicated by Fig. 3(d1), the dividing lines *I*_*PF1*_/*I*_*PF2*_ = 1.3 and 2.5 of three situations are sketched as green dash lines. The probabilities of the situations A–C are 2.4%, 70.8% and 26.8%, respectively. Moreover, the random changes of multiple filamentation corresponding to the second pair hot spots (filament 3 and 4) are shown in the Supplementary Movie 2. The three different situations of the two filaments in the movie 2 at three distinct times are also depicted accordingly in Fig. 3(a2–c2). Similarly, the statistical analysis of the filamentation competition events is depicted in Fig. 3(d2), where *I*_*PF3*_ and *I*_*PF4*_ are the higher peak fluorescence signal intensity and the other one of the filaments 3 and 4, respectively. The probabilities of situation A–C are 39%, 58.6% and 2.4% for the second pair filaments, respectively. It can be also seen from the Supplementary Movie 2 and Fig. 3(a2–c2) that the refocusing peaks in a single-shot pulse is appear for the hotter spot, which is the result of the interplay of self-focusing and plasma defocusing for a relative high pulse energy. This is one of the important physical mechanisms of the long-range propagation and filament formation in optics media^32^.

**Figure 4:**
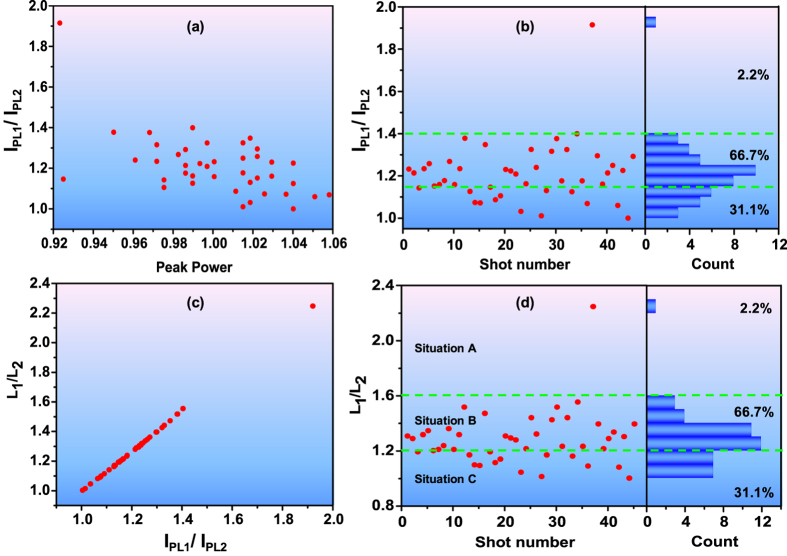
(**a**) The relation between the peak laser intensity ratio of the hot spots and the peak power of input pulses. (**b**) The statistic analysis of the jitter of the peak laser intensity ratio of the hot spots corresponding to the filaments 1 and 2. (**c**) The relation between the ratio of the start locations of filamentation and the peak laser intensity ratio of the hot spots. (**d**) The probabilities of situations A–C calculated theoretically by the simplified (2D+1)-dimensional model based on (**a**).

The detected irregular changes of the two pairs of filaments from shot to shot can be explained by fluctuation arising from the input pulses itself. Though the locations of the hot spots are controllable and stable, which are demonstrated by the almost invariable locations of multiple filaments for all shots, the pulse energy transmits through the narrow iris1 is sensitive to the perturbations of the power and distribution of femtosecond laser. So that the intensity and its ratio of the two hot spots corresponding to the two chosen filaments still have a remarkable change for different pulses. This can severely affect the field redistributed inside the pulse and lead to the randomly changes for different shots as the result of the competition for energy from background energy reservoir in the filamentation process. In order to clarify the physical mechanism of the irregular multiple filaments of different pulses, the filaments 1 and 2 and the corresponding hot spots are discussed more detail for conciseness and without loss of generality. The relation between the peak laser intensity ratio of hot spots and the peak power of input pulses is shown in Fig. 4(a), where the higher peak laser intensity and the other one of hotspots 1 and 2 are denoted as *I*_*PL1*_ and *I*_*PL2*_, respectively, the peak power of input pulses is normalized by the mean value of all the shots. Even though the jitter of peak power after narrow iris1 (~8%) is greater than the initial laser power fluctuation (<3%), it is not remarkable comparing with the jitter of the peak laser intensity ratio *I*_*PL1*_/*I*_*PL2*_ of the hot spots and the intensity ratio is not related to the fluctuation of the laser power at all. As a consequence, the jitter of the peak laser intensity ratio of the hot spots corresponding to the filaments 1 and 2 is focused and statistically analyzed in Fig. 4(b). It can be seen that the probability distribution of the peak laser intensity ratio of the hot spots is similar to the ratio of the maximum fluorescence signal intensity between filaments 1 and 2. Then the numerical simulations will be performed to quantitatively demonstrate how the jitter of the peak intensity ratio of the hot spots can lead to the random changes of multiple filamentation.

Note that the statistics of 40 laser shots has been analyzed in our case. It is well known that the larger sample sizes generally lead to increased precision when estimating unknown parameters. In some situations, the increase in precision for larger sample sizes is minimal, or even non-existent so that the specific sample sizes is determined by the factors such as Confidence interval and errors. There is a sample formulation to estimate sample size $N={u}_{\alpha }^{2}P(1-P)/{\delta }_{e}^{2}$, where *N* is the sample size,*u*_*α*_ is determined by the confidence coefficient, *P* is the probability and *δ*_*e*_ is the allowable error. In our experiment, if we adopt the situation B to estimate the sample sizes and the confidence coefficient and allowable error are set at 90%(${u}_{\alpha }=1.28$) and 10%, respectively, the corresponding sample size is 35. It implied that the shot number in our experiment is enough for the statistic analysis which mainly care about the three situations.

### Numerical simulations

All of above observations provide a physical scenario that the interaction between two filaments includes independent development from the initial perturbations; redistribution of the laser energy between all the filaments resulting in the survival and disappearance of the other one; fusion each other when their energy is almost the same. To clarify the physical mechanism for the irregular multiple filaments for different pulses under the condition that the locations of the hot spots are stable for the input beam, numerical simulations based on a (2*D*+1)-dimensional nonlinear wave equation have been carried out:

1 $$2i{k}_{0}\frac{\partial A}{\partial z}+(\frac{{\partial }^{2}}{\partial {x}^{2}}+\frac{{\partial }^{2}}{\partial {y}^{2}})A+2{k}_{0}^{2}{\rm{\Delta }}nA=0$$

where *A* represents the amplitude of the light field, *k*_0_ denotes the wave number of the beam whose central wavelength is 800 nm. *Δn* = *n*_2_ *I* − *σI* ^*m*^ is the nonlinear refractive index originating from the optical Kerr effect induced nonlinear refractive index (*n*_2_ *I*) and an effective counteracting higher order nonlinear refractive index (−*σI* ^*m*^), where *n*_2_ = 1.67 × 10^−16^ cm^2^/W corresponding to a critical power of 4 MW^33^, *m* is chosen to be equal to 5, which is approximately the reported effective nonlinearity order in methanol by near infrared femtosecond laser. *σ* denotes an empirical parameter, which gives rise to a clamped intensity of 1 × 10^13^ W/cm^2^,^32^. To model the intensity and its ratio of the two hot spots, the initial distribution of the electric field complex amplitude was taken as the superposition of the two Gaussian functions

2 $$A(x,y,z=0)={A}_{1}\,\exp [-\frac{{x}^{2}+{(y-{y}_{1})}^{2}}{{{r}_{1}}^{2}}]+{A}_{2}\,\exp [-\frac{{x}^{2}+{(y-{y}_{2})}^{2}}{{{r}_{2}}^{2}}]$$

*A*_1,2_, (0, *y*_1,2_), *r*_1,2_ are the amplitude, position, and radius of the first (second) Gaussian beam. The distance *d* between them is

3 $$d=|{y}_{1}-{y}_{2}|$$

The input two Gaussian beams with invariable peak power but different peak intensity ratios are considered in our simulation to verify the analysis above. The radius *r* (1/*e* ^2^) of each Gaussian beam is 0.1 mm and the distance between them is 0.2 mm.

**Figure 5:**
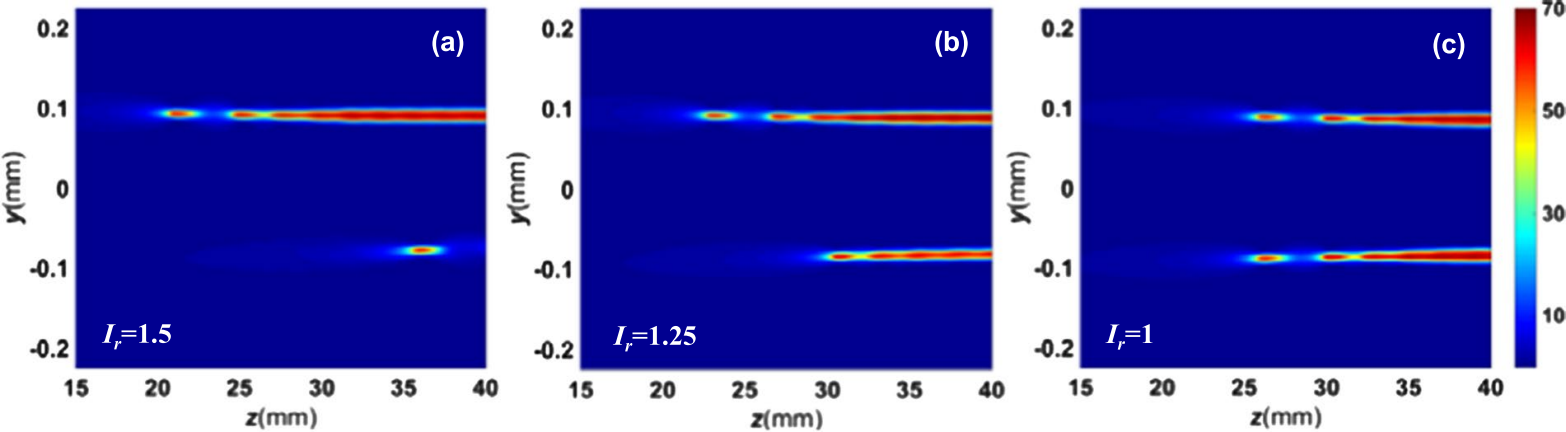
(**a**–**c**) The simulated longitudinal laser intensity distributions in the *x* = 0 plane corresponding to the input two Gaussian beams with the peak intensity ratios of 1.5, 1.25 and 1, respectively.

Based on the jitter of the peak laser intensity ratio of hot spots shown in Fig. 4(b), the simulated longitudinal laser intensity distributions in the *x* = 0 plane corresponding to the input two Gaussian beams with the peak intensity ratios *I*_*r*_ of 1.5, 1.25 and 1 are depicted in Fig. 5(a–c), respectively. The peak intensities are all normalized by *I*_0_, where *I*_0_ = 10*P*_*cr*_/*πr* ^2^ and *P*_*cr*_ is the critical power of methanol. It can be observed that the numerical simulations can qualitatively reproduce the three situations of experimental results. Specifically speaking, Fig. 5(a) corresponds to Fig. 3(a) in which one filament almost disappear, Fig. 5(b) corresponds to Fig. 3(b) in which one filament is immature, Fig. 5(c) corresponds to Fig. 3(c) in which the two filaments are comparable to each other. Furthermore, to quantitatively demonstrate the reliability of this theoretical explanation and considering the probability histograms of Figs 3(d1) and 4(b) and the simulated longitudinal laser intensity distributions in Fig. 5(a–c), the scattered points in Fig. 4(b) can be also divided into three situations just as Fig. 3(d1) and the dividing lines of three situations are *I*_*PL1*_/*I*_*PL2*_ = 1.15 and 1.4, which are sketched as green dash lines. Based on the jitter of the peak laser intensity ratio of the hot spots in Fig. 4(b) and the simplified (2D+1)-dimensional model, the relation between the ratio of the start locations of filaments (*L*_1_/*L*_2_) and the peak laser intensity ratio of the hot spots (*I*_*PL1*_/*I*_*PL2*_) is depicted in Fig. 4(c). The vertical axis *L*_1_/*L*_2_ is the ratio of the start locations of filaments. The start location of filament is determined by the hotspot energy and represents the intensity of filament. It can be seen that the only one point of *I*_*PL1*_/*I*_*PL2*_ = 1.92 produced a rare filamentation competition event, that is the Situation A in Fig. 3(d1). Moreover, the probabilities of filamentation competition events of other situations can be also calculated theoretically as shown in Fig. 4(d), which are 31.1%, 66.7% and 2.2% for the three situations corresponding to situations A–C in Fig. 3(d1), respectively. The green dash dividing lines are *L*_1_/*L*_2_ = 1.2 and 1.6 which correspond to *I*_*PL1*_/*I*_*PL2*_ = 1.15 and 1.4 in Fig. 4(b). In a word, the statistical results of the three situation of filamentation competition can be quantitatively reproduced by combining numerical simulation with the experimental results of input beam. That is, the random change of the visible filaments from shot to shot can be well explained by the peak intensity ratio perturbation of the input beam.

Note the dividing lines are objectively chosen in both Fig. 3(d1) and Fig. 4(b) with similar probability in order to demonstrate the crucial role of the hot spots power ratio on the multiple filament competition. For the sake to confirm this point, Fig. 4(c) has been depicted, whose horizontal axis represents the hot spots power ratio and vertical axis denotes the ratio of the starting point of the two filaments in the simulation. Worth mentioning that compared with the experiments, some detailed effects, including temporal evolution of the pulse, fluorescence excitation, have been neglected in our numerical simulations. In addition, the input laser hot spots have been assumed to be Gaussian. Therefore, apart from the fluorescence signal ratio used in the experiment, the ratio of the starting position of two filaments has been evaluated as the criteria of the competition. Figure 4(c) displays strong correlation between the ratio of the starting position of two filaments and the power ratio of two hot spots, which could not be explained by the simple power dependence of the self-focusing distance.

## Conclusion

In conclusion, the unique 3-photon florescence technique was performed to realize the visual observation of the multiple filamentation competition phenomena in a single-shot pulse propagating in liquid methanol dissolved with coumarin 440. The expected location controllable multiple hot spots were obtained by utilizing straight-edge diffraction and spatial filtering. It can be found that the random change of the visible filaments from shot to shot which can be explained by the peak intensity ratio perturbation of the hotspots in the input pulses. Moreover, the explanation was elaborately demonstrated by the simplified numerical simulation. The visual observation of the multiple filaments provides insight into the understanding of the origin of multiple filaments, consequent propagation, and interaction dynamics which is critical to the control of multiple filaments and the various applications of filamentation.

## Electronic supplementary material

Supplementary Movie 1

Supplementary Movie 2
